# Feasibility of Assessing Diet with a Mobile Food Record for Adolescents and Young Adults with Down Syndrome

**DOI:** 10.3390/nu9030273

**Published:** 2017-03-13

**Authors:** Katherine E. Bathgate, Jill L. Sherriff, Helen Leonard, Satvinder S. Dhaliwal, Edward J. Delp, Carol J. Boushey, Deborah A. Kerr

**Affiliations:** 1 School of Public Health, Curtin University, Perth, WA 6845, Australia; katherine.bathgate@postgrad.curtin.edu.au (K.E.B.); j.sherriff@curtin.edu.au (J.L.S.); S.Dhaliwal@curtin.edu.au (S.S.D.); 2 Telethon Kids Institute, University of Western Australia, West Perth, WA 6872, Australia; Helen.Leonard@telethonkids.org.au; 3 School of Electrical and Computer Engineering, Purdue University, West Lafayette, IN 47907, USA; ace@ecn.purdue.edu; 4 Epidemiology Program, University of Hawaii Cancer Center, Honolulu, HI 96813, USA and the Nutrition Program, Purdue University, West Lafayette, IN 47907, USA; CJBoushey@cc.hawaii.edu

**Keywords:** mobile food record, novel technology, dietary assessment, young adults, adolescents, Down syndrome

## Abstract

Technology-based methods for assessing diet in those with disability remains largely unexplored. The aim was to assess the feasibility of assessing diet with an image-based mobile food record application (mFR) in 51 adolescents and young adults with Down syndrome (PANDs). Adherence was also assessed with the instruction to include a fiducial marker object in the before and after eating images. The PANDs sample completed a four-day mFR and results were compared with a sample of young adults from the Connecting Health and Technology study (CHAT, *n* = 244). Compared to the CHAT sample, PANDs participants reported more fruit (2.2 ± 1.8 versus 1.0 ± 0.9 serves respectively) and vegetables (2.4 ± 1.3 versus 1.9 ± 1.0 serves, respectively), but no differences in energy-dense nutrient-poor (EDNP) foods and beverages were observed. Compared to CHAT, PANDs participants captured fewer images with the mFR (4.9 ± 2.3 versus 4.0 ± 1.5 images, respectively). Adherence to the instruction to include the fiducial marker in images was lower for PANDs compared with the CHAT sample (90.3% versus 96.5%). Due to the quality of information captured in images and the high acceptability of the fiducial marker, the mFR shows great promise as a feasible method of assessing diet in adolescents and young adults with Down syndrome.

## 1. Introduction

In Western Australia (WA), the prevalence of intellectual disability is 17 in 1000 live births, with Down syndrome being the most common biomedical cause accounting for 6.2% of all cases [1]. Down syndrome is associated with varying levels of intellectual disability as well as an increased risk of cardiac, gastrointestinal and endocrine disorders, orthopaedic conditions, hearing and sight impairments, dementia and obesity [2]. Due to advancing medical and social advancements, life expectancy for people with Down syndrome is increasing [3], highlighting the importance of addressing chronic disease risk factors such as sub-optimal dietary intake [4]. In the 2011–2013 Australian Health Survey, over a quarter of adolescents aged 12–17 years and over a third of young adults aged 18–24 years were overweight or obese [5]. Published data on the prevalence of overweight and obesity in adolescents and young adults with Down syndrome indicate even higher levels of overweight and obesity [6,7,8,9]. In a large (*n* = 197) population-based Western Australian study, 57.4% of adolescents and young adults with Down syndrome aged 16–30 years were perceived by their parents and carers to be overweight or obese [10].

There are limited data on what young adults with intellectual disabilities are eating due to the challenges of assessing diet in this population [4,11,12]. Acceptable dietary assessment methods for people with intellectual disabilities present additional challenges due to difficulties with memory, cognition, literacy and communication [4]. Previous studies with adolescents and young adults with Down syndrome have utilised a variety of dietary assessment methods [13,14,15,16]. In almost all these studies, parents and carers completed the questionnaires on behalf of the young person with Down syndrome [13,14,16], with few studies involving the young people themselves in data collection [15]. For adolescents and young adults, their growing independence outside the home can limit the acceptability of proxy-reported dietary intake [4]; thus, for adolescents and young adults with Down syndrome, mobile technologies may improve participation in dietary intake research.

Mobile devices such as mobile telephones are used by people with intellectual disabilities [17]. Although early research suggested that the use of mobile phones by people with intellectual disabilities was less than by people without intellectual disabilities [18], more recent research has found increasing use of mobile phones by adults with intellectual disabilities [17]. Studies investigating the use of mobile devices such as iPods and iPads demonstrate people with intellectual and developmental disabilities can manipulate these devices and they enjoy using them [19,20,21]. The use of image-based dietary assessment has several advantages over written food records [22]. Images taken in real time can provide accurate information on the type and amount of food and beverages consumed whilst potentially reducing the recording burden associated with written food records for participants [22,23]. An image-based dietary assessment system known as Technology-Assisted Dietary Assessment or TADA [24,25,26,27] uses the camera on a mobile device to capture before and after images of food and beverages consumed. Referred to as the mobile food record (mFR) app, the participant is instructed take a before and after image of all food and beverages and to include a reference device known as a fiducial marker (a checkerboard pattern of known shape, size and colour) to assist with food identification and portion size estimation [26,28]. Although trialled with children [29], adolescents [30] and young adults without disabilities [31], it has not been trialled with people with intellectual disabilities. The aim of this research was to assess the feasibility of assessing diet with an image-based mobile food record application in adolescents and young adults with Down syndrome. A second objective was to assess the acceptability of the use of the fiducial marker in this population.

## 2. Materials and Methods

### 2.1. Study Design

This study was a cross-sectional analysis of food and beverage intake captured using the mobile food record from two studies—the Physical Activity, Nutrition and Down syndrome (PANDs) study and the Connecting Health and Technology (CHAT) study previously published [31,32]. The PANDs study was a cross-sectional study of body composition, dietary intake and physical activity of adolescents and young adults with Down syndrome. Approval for both studies was granted by the Curtin University Human Ethics Research Committee (HR145/2011, HR181/2011) and the CHAT trial was registered (Australian Clinical Trials Registry Registration number ACTRN12612000250831). Approval for the PANDs study was also obtained from the Disability Services Commission (WA).

### 2.2. Participant Recruitment

#### 2.2.1. PANDs Study

Participants with Down syndrome aged 12–30 years living within a 250 km radius of Perth, Western Australia, were recruited from the Down syndrome NOW (Needs, Opinions, Wishes) database [33] and contacted by mail. Informed consent was obtained from a parent/guardian and where possible, from the participant providing the individual was at least 18 years of age. If the latter consent was not possible then assent from the young person was obtained before proceeding. Assent was also required from children younger than 18 years of age. There were no exclusion criteria.

#### 2.2.2. CHAT Study

Young adults (18–30 years) living in the Perth metropolitan area were recruited via the Federal Electoral Roll and contacted by mail. Details of the study protocol and outcomes have been previously published [31,32]. Participants were excluded if they were unable to attend the study site over the six-month study period, were following a restrictive diet, participating in extreme exercise, studying nutrition at University, or were pregnant or breastfeeding or if they had any serious illnesses. Of the 247 recruited, three participants were excluded for this analysis due to incomplete (less than one image-pair per day) mobile food records.

### 2.3. Data Collection

Participants in both studies completed a four-day mobile food record (mFR) using an Apple iPod Touch. Height and weight were measured for participants in both studies following standard procedures [34]. In both studies, participants underwent the same training on how to use the mFR app for the collection of dietary information. The training session included information on how to: connect to Wi-Fi for sending images; take a practice image of plastic food replicas; and sending the before and after image pair to the back-end server. Participants were instructed to record their food and beverage intake using the mFR app for four consecutive days (including one weekend day) with the investigator-supplied iPod Touch (iOS6, Apple Inc., Cupertino, CA, USA) loaded with the mFR app. When taking an image, participants in both studies were instructed to include a reference device known as a fiducial marker to assist with food identification and portion size estimation. They were instructed to record food and beverage items not captured using the iPod notes section or in a small booklet provided that also contained visual and written instructions on using the mFR app. For both studies, on returning the iPod one week later, a dietitian confirmed the contents of the images and probed for any forgotten recordings with participants.

#### 2.3.1. PANDs Study

**Figure 1:**
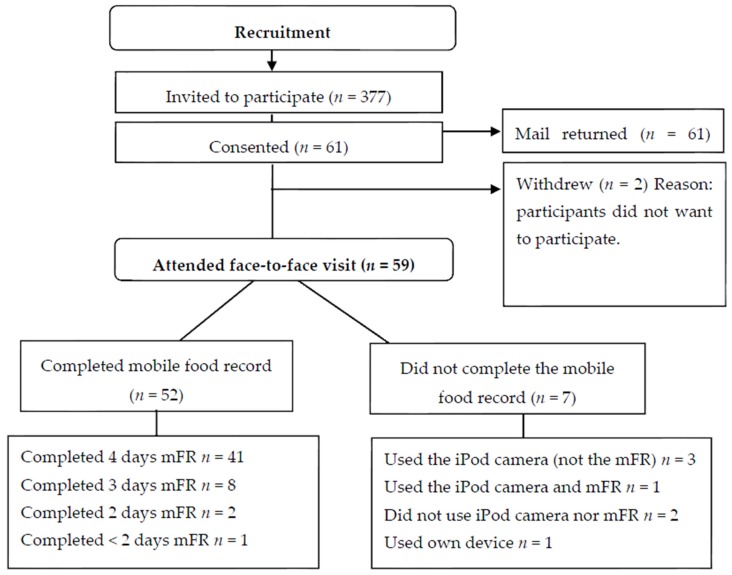
Data collection flowchart for the PANDs study.

Participants in the PANDs study along with their family members/carers were visited by the research dietitian in their own home or a venue of their choosing and trained in the use of the iPod and the mFR app. The training consisted of physical and verbal instruction in the use of the iPod and mFR app, including the capturing of practice food and beverage images, firstly with the participants followed by their family members/carers. Family members/carers were reassured that connection to Wi-Fi was not essential and images could be uploaded securely once the device was returned. Participants were encouraged to carry the iPod with them at all times however the iPods could not be taken to schools and some family members and carers were hesitant about the iPod going to work, day activities or social occasions. Therefore if the participant with Down syndrome took food and beverages with them to school, work or other activities, it was recommended that images of the food and beverages were captured at home beforehand or notes made in the booklet. The PANDs study dietitian reviewed the images and also took a diet history to capture usual diet and daily activities involving both the participant and the family members/carers who accompanied them. This was done to confirm the contents of the images that were consumed or prompt identification of any missing foods or beverages not captured using the mFR app or recorded in the paper booklet. Participants in the PANDs study received a recipe book donated by the Health Department of WA. Figure 1 shows the data collection flow for the PANDs study; data flow for the CHAT study has been previously described [31,32].

#### 2.3.2. Image Analysis in the PANDs Study

**Figure 2:**
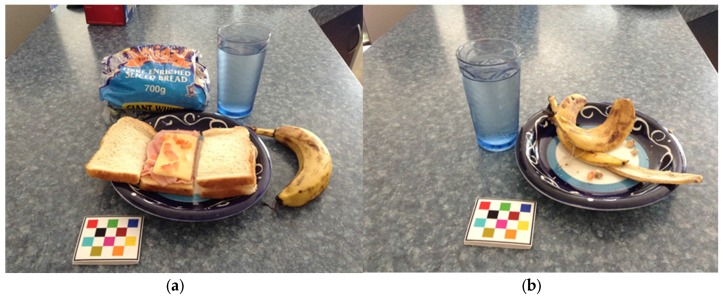
Example of the image capture by a PANDs participant using the mFR. (**a**) Before eating image; (**b**) After eating image. The image also shows the inclusion of the fiducial marker in the images. The loaf of bread was included in the image to help with identification of the type of bread consumed, only what was on the plate was analysed.

Food and beverage images from the mFR and fiducial marker inclusion in the images were assessed by two trained analysts. An example of a before and after image is shown in Figure 2. Each before and after image was recorded as an image pair. To be included in the food and beverage analysis (Table 1), a minimum of two full days’ food record needed to be completed (either using the mFR app, iPod camera without using the mFR app or written food record). The procedure for the analysis of the mFR was the same for both studies. The research dietitian assessed the mFR food and beverage images and used a quality scoring of food items by food group serves sizes according to the Australian Guide to Healthy Eating (AGHE) [35]. A purpose-built Microsoft Access data table was developed for food and beverages data entry with linked categories for food group, food type and serving size. For each participant, an average serve per day was calculated for fruits, vegetables, sugar sweetened beverages (SSB), energy-dense, nutrient-poor (EDNP) foods and alcohol. Acceptability of the fiducial marker was determined by analysing the presence or absence of the fiducial marker in all images captured using the mFR. The fiducial marker was either recorded as present, partially present, absent or OOPS. Partially present was where only part of the fiducial marker was visible in the image, with the rest of the marker either outside the image or obscured in some way. OOPS referred to when the participant or their family member/carer had not taken an after image of the food or beverage consumed. They were instructed to capture an image of the word OOPS (which was written on the reverse of the fiducial marker) to replace the after image. The mFR app had an inbuilt reminder prompting participants if they had forgotten to take an after image. If participants ignored this reminder and proceeded with taking their next before image, this image was captured in place of an after image. This situation occurred with some of the PANDs participants.

### 2.4. Statistical Analysis

The statistical package used for all analysis was SPSS Statistics v. 24 (IBM Corp., Armonk, NY, USA, 2016). Descriptive statistics were used to describe and compare participant characteristics, food group serves and mFR images. Differences in the mean of food group serves, number of image pairs, height, weight and body mass index (BMI) in both studies were compared in total and by gender using independent *t*-tests. Statistical significance was set at *p <* 0.05.

## 3. Results

In the PANDs study, of the 377 adolescents and young adults who were invited to participate, consent was provided for 61 participants (Figure 1). Two participants withdrew from the study as they chose not to participate, resulting in a 15.6% response fraction. Of the 59 participants, 52 completed the study using the mFR app, six did not use the mFR app and one participant used a combination of the mFR app and images taken using the iPod camera. Another participant who used the mFR app did not complete two full days of recording and their data have been excluded from the results, with 51 participants using the mFR app for a minimum of two days.

The physical characteristics of all participants in both studies are shown in Table 1. Participants in the PANDs study were younger on average than participants in the CHAT sample (21.5 ± 4.6 years vs. 24.3 ± 3.4 years, *p* < 0.001); however, this was expected as the target cohort age was younger (12–30 years compared to 18–30 years). Participants in the PANDs study had a higher mean BMI, especially among females, and a lower mean height compared to participants in the CHAT sample (*p <* 0.001).

When comparing food group intake between all participants of the two studies (including PANDs participants who did not use the mFR app), there was no difference in the mean number of daily serves of sugar-sweetened beverages reported; however, participants in the PANDs study reported a higher number of serves of fruit (2.2 ± 1.8 vs. 1.0 ± 0.9) and vegetables (2.4 ± 1.3 vs. 1.9 ± 1.0) compared with participants in the CHAT study (see Table 1). Women in the PANDs study reported fewer daily serves of EDNP foods than female participants in the CHAT sample (2.4 ± 1.2 vs. 3.1 ± 1.5, *p* = 0.015). This outcome was not seen in males or in the groups as a whole. Comparing adults in the PANDs study (*n* = 45) to those in the CHAT sample, there continued to be a higher mean number of serves of fruit reported by both genders and a lower mean number of serves of EDNP foods reported by women in the PANDs study; however, a significant difference in the mean number of serves of vegetables reported was not observed in females. 

Table 2 shows the use of the mFR app and the inclusion of the fiducial marker in the images. In those participants who used the mFR app there was a difference in the mean number of image pairs from the participants in the PANDS study compared to participants in the CHAT sample (4.0 ± 1.5 vs. 4.9 ± 2.3, *p <* 0.01) and differences in the inclusion of the fiducial marker in the images (see Table 2). There was less inclusion of the fiducial marker in before images for the PANDs study with 6.7% of images missing the fiducial marker, compared to 2.1% of images in the CHAT sample. In the PANDs study there were also 11 more before images compared to the after images (see Table 2). This was due to an after image not being captured and a before image of the next meal or snack being captured in its place.

## 4. Discussion

In this study, adolescents and young adults with Down syndrome were able to capture images of their diet using a mobile food record, with 86% (51 of 59) of participants collecting dietary intake for at least two full days. Fewer image pairs were captured using the mFR app by participants with Down syndrome and their families/carers compared to participants without Down syndrome. Nevertheless, the acceptability of the fiducial marker among participants with Down syndrome was high with the marker fully present in 88.9%–90.3% of images captured using the mFR app. This use of the fiducial marker is higher than previous research using a poorly constructed fiducial marker with adolescents (*n* = 18) without disability [36]. This higher rate in the present study may have been assisted by the in-built technology of the mFR version used in this study that prompts the user to include the marker if not detected [26,28]. The mFR app used in the current study appears to be a feasible dietary assessment method for adolescents and young adults with Down syndrome.

For the seven participants and their families who did not use the mFR app, the most popular method of data collection was the capturing of images using the iPod camera or another mobile device camera, with two families providing written food records in place of images. This could be due to some participants being less familiar with the iPod device or reluctance to use the mFR app to take images in public or at work. This is consistent with findings in a study with young adults with intellectual disabilities where diet was captured with an image-assisted recall method [12]. This finding also highlights the importance of the initial training for young people with Down syndrome and their families/carers in using the mFR app and suggests that improvements such as video instructions preloaded onto the iPod could be of assistance in future studies.

Participants in the PANDs study reported a significantly greater number of servings of fruit and vegetables compared to participants in the CHAT study. Similar findings were also observed in two studies of adults with Down syndrome (both *n* = 51) where, compared to controls, adults with Down syndrome reported a greater mean intake of fruit and vegetables [37,38]. Earlier research with overweight and obese adolescents (*n* = 61) in WA found low average intakes of fruit (0.7 serves) and vegetables (1.2 serves) [39]; however, these low intakes were not observed in the PANDs study. These observed differences could be due to family members having more positive influences on fruit and vegetable intake for the PANDs participants, compared to other adolescents and young adults. However, there were no differences in the consumption of energy-dense nutrient-poor foods and sugar-sweetened beverages between the PANDs and CHAT study participants, except for a lower consumption of EDNP foods in females with Down syndrome. 

The strengths and limitations of the CHAT study have been discussed previously [31]. The PANDs study sample was representative of the gender distribution of people with Down syndrome in WA, with 56.6% of infants born from 1980–1996 being male [40], similar to 53% of the PANDs study sample being male. As with previous studies using iPads with adolescents and young adults with intellectual disabilities [11,12], the PANDs study involved the young person with Down syndrome as much as possible in the collection of food intake data, with participants trained and encouraged to use the mFR app themselves. The mFR app also has several advantages over simply using a mobile device camera. The mFR app allows images to be uploaded to a secure server and not stored on the device. This ensured the images could not be accidentally deleted even if the mFR was deleted. The mFR images also include a time and date stamp. The requirement (including built-in prompting) to take both a before and after image ensured that any uneaten portion was captured and could be taken into account during analysis. In addition, the mFR app indicated if the fiducial marker was not in the image, prompting another image to be taken, and immediate feedback through the use of a colour border indicated if the angle of the photograph was optimal (green) or not (red) for analysis.

The PANDs study had several limitations. The number of images taken by the participants with Down syndrome rather than the family members or carers was not recorded. Previous studies using the mFR app have demonstrated the importance of user feedback [41,42] but this was not undertaken in the PANDs study. User feedback may have been helpful in identifying issues, such as identifying the reasons for the 11 extra before images taken by PANDs participants. It is recommended future studies in participants with Down syndrome include user feedback, to better inform the assessment of diet using the mFR app. Additionally, the measurement error from unrecorded foods and beverages or changes to usual dietary patterns could have influenced the results [43,44]. There may have been a social desirability bias in those who participated in the PANDs and CHAT studies but this was not assessed in either study [45]. The families of participants who volunteered to participate in the PANDs study may have been more interested in nutrition, and families who declined the invitation were not surveyed as to their reason for refusal. Families who lived further than 250 km from the Perth metropolitan region were not invited due to travel constraints, and these selection biases [46] may have impacted the results. In both the CHAT and PANDs studies, the identification of food and beverages and the estimation of portion size was undertaken by different trained analysts and therefore there may be differences in estimation. Using the camera function on a mobile device such as an iPod may have been more familiar to PANDs study participants if they had previously used these devices. Compared to using the iPod camera, the mFR app required additional steps in taking the image (e.g., turning the iPod horizontally to capture the image) and buttons on the screen had written instructions (e.g., ‘snap it’) [27]. The need to read instructions was compensated for, however, by the buttons always being in the same place on the screen, and participants with Down syndrome and their family members/carers receiving training on how to use the app and device, and written and visual instructions being left with participants and families. This training could have been enhanced by the development of a step-by-step video loaded on the iPod to which participants and their families could refer. The mFR application was also accidentally deleted from the iPod by one participant. In the current study, the mFR application was provided on the study iPod. With advances in technology, there is now the capability to install the mFR application on the participants’ own devices which may improve the acceptability in future studies. 

## 5. Conclusions

The mFR is a feasible app for adolescents and adults with Down syndrome. The mFR app was used by most participants and their families in the PANDs study to record foods and beverages consumed, providing detailed visual information and reducing the burden of traditional food intake data collection. In future studies, the development of video instructions, accessible on the iPod, could better assist participants with Down syndrome in using the mFR app, further enabling young people with Down syndrome to be involved in the collection of their own dietary intake data. Future studies should also evaluate the ease of use of the mFR app by adolescents and young adults with Down syndrome and suggest ways to improve the usability if required.

## Figures and Tables

**Table 1:** Characteristics of participants in the (Physical Activity, Nutrition and Down syndrome) (PANDs ^1^) and Connecting Health and Technology (CHAT) studies who completed a food record using the mobile food record (mFR) app.

	PANDs Study						CHAT Study					
	Male *n =* 31 (53%)		Female *n =* 27 (47%)		Persons ^2^ *n =* 58 (100%)		Male *n =* 82 (34%)		Female *n =* 162 (66%)		Persons *n =* 244 (100%)	
Characteristics	Mean	SD	Mean	SD	Mean	SD	Mean	SD	Mean	SD	Mean	SD
Age (year)	21.7 ***	4.6	21.3 ***	4.7	21.5 ***	4.6	24.7 ***	3.4	24.1 ***	3.4	24.3 ***	3.4
Height (cm)	158.0 ***	6.3	145.8 ***	6.8	152.3 ***	9.0	178.3 ***	7.3	165.0 ***	6.8	169.5 ***	9.4
Weight (kg)	65.9 ***	12.7	61.3	15.5	63.8 **	14.2	78.7 ***	15.3	65.6	15.6	70.0 **	16.7
BMI	26.4	4.7	28.6 ***	6.5	27.4 ***	5.7	24.7	4.4	24.1 ***	5.8	24.3 ***	5.4
**Food group serves**												
Fruit daily serves (150 g)	2.3 ***	1.9	2.0 ***	1.6	2.2 ***	1.8	1.1 ***	1.3	0.9 ***	0.7	1.0 ***	0.9
Vegetable daily serves (75 g)	2.4 *	1.5	2.4 **	1.1	2.4 ***	1.3	1.9 *	1.0	1.9 **	1.0	1.9 ***	1.0
EDNP ^3^ daily serves (600 kJ)	3.2	1.7	2.4 *	1.2	2.8	1.5	3.3	2.0	3.1 *	1.5	3.2	1.7
SSB ^4^ daily serves (600 kJ)	0.5	0.7	0.4	0.4	0.4	0.6	0.5	0.7	0.5	0.6	0.5	0.6

^1^ For PANDs data includes participants who completed the food record using either the mFR app, the iPod camera, a written food record or a combination for a minimum of two days; ^2^ Statistical comparisons are between studies for males, females and persons; ^3^ Total energy-dense nutrient-poor (EDNP) food group serves includes junk foods, sugar-sweetened beverages (SSB) and alcohol; ^4^ sugar-sweetened beverages; *, **, *** Significantly different by independent sample t-test at *p <* 0.05, *p <* 0.01 and *p <* 0.001 respectively, SD = standard deviation.

**Table 2:** Use of the mobile food record (mFR) app and inclusion of the fiducial marker in images taken by participants in the Physical Activity and Down syndrome (PANDs) and Connecting Health and Technology (CHAT) studies.

	PANDs Study (*n* = 51)		CHAT Study (*n* = 244)	
	*N* before Images (%)	*N* after Images (%)	*N* before Images (%)	*N* after Images (%)
Fiducial marker present	692 (90.3%)	671 (88.9%)	4768 (96.6%)	4528 (91.8%)
Fiducial marker partially present	23 (3.0%)	24 (3.2%)	12 (0.2%)	17 (0.3%)
Fiducial marker missing	51 (6.7%)	58 (7.7%)	105 (2.1%)	110 (2.2%)
OOPS ^1^	0	2 (0.0%)	50 (1.0%)	280 (5.7%)
Total	766 (100%)	755 (100%) ^2^	4935 (100%)	4935 (100%)

^1^ OOPS. Participants were instructed to include an image of the OOPS on the alternate side of the fiducial marker when they had forgotten to take an after image; ^2^ Difference in totals was due to a before image of the next meal or snack being captured in place of an after image.
